# Residual inflammatory risk and vulnerable plaque in the carotid artery in patients with ischemic stroke

**DOI:** 10.3389/fneur.2024.1325960

**Published:** 2024-04-24

**Authors:** Xiuqun Gong, Chuanqing Yu, Zeyu Lu, Xia Wang, Qiankun Cai, Xiaosi Cheng, Jun Lu

**Affiliations:** ^1^Department of Neurology, The First Affiliated Hospital of Anhui University of Science and Technology, The First People's Hospital of Huainan, Huainan, Anhui, China; ^2^Stroke Center and Department of Neurology, The First Affiliated Hospital of USTC, Division of Life Sciences and Medicine, University of Science and Technology of China, Hefei, Anhui, China; ^3^Department of Clinical Laboratory, Taihe Hospital Affiliated of Wannan Medical College, Taihe County People's Hospital, Fuyang, Anhui, China; ^4^Department of Neurology, The Second Affiliated Hospital of Fujian Medical University, Quanzhou, Fujian, China; ^5^Department of Medical Laboratory, College of Medicine, Anhui University of Science and Technology, Huainan, Anhui, China

**Keywords:** inflammatory risk, atherosclerosis, vulnerable plaque, carotid artery, ischemic stroke

## Abstract

**Objective:**

Inflammation is a central driver of atherogenesis and eventual plaque rupture. This study aimed to evaluate the association between residual inflammatory risk (RIR) and vulnerable plaques in the carotid artery in patients with ischemic stroke.

**Methods:**

Patients with acute ischemic stroke were enrolled from January 2021 to July 2022. They were divided into four groups: RIR only (LDL-C <2.6 mmol/L and hsCRP ≥2 mg/L), residual cholesterol risk (RCR) only (LDL-C ≥2.6 mmol/L and hsCRP <2 mg/L), both risk or residual cholesterol and inflammatory risk (RCIR) (LDL-C ≥2.6 mmol/L and hsCRP ≥2 mg/L), and neither risk (LDL-C <2.6 mmol/L and hsCRP <2 mg/L). Vulnerable plaques were determined if it had a low attenuated plaque CT value of <35 Hounsfield Units (HU) and a remodeling index of >1.1, which indicated a positive remodeling.

**Results:**

Out of the 468 enrolled patients, 157 (33.5%) were detected to have vulnerable plaques. The proportion of patients with neither risk, RIR, RCR, and RCIR were 32.9%, 28.6%, 18.8%, and 19.7%, respectively. Patients with vulnerable plaques exhibited a higher prevalence of hyperlipidemia (*P* = 0.026), higher proportion of RIR (*P* = 0.015), a higher ratio of stroke subtypes of large artery atherosclerosis (*P* = 0.012), and high leukocyte counts (*P* < 0.001). The logistic regression analysis detected that RIR was associated with vulnerable plaques after adjusted for major confounding factors (OR 1.98, 95% CI 1.13–3.45, *P* = 0.016), especially in the large artery atherosclerosis subtype (OR 2.71, 95% CI 1.08–6.77, *P* = 0.034).

**Conclusions:**

In patients with ischemic stroke, RIR is associated with the vulnerability of carotid plaques, especially for those with the large artery atherosclerosis subtype. Therefore, further studies investigating the interventions to modulate inflammation in these patients may be warranted.

## Introduction

Acute ischemic stroke is the most important subtype of stroke and is also one of the major causes of economic burden in China (1). Approximately one fifth of the ischemic stroke is associated with atherosclerosis of the carotid arteries (2). Generally, atherosclerotic plaques can be divided into stable plaques and unstable or vulnerable plaques. Research studies have shown that whether carotid atherosclerotic plaque leads to ischemic stroke is mainly determined by whether the plaque is stable, rather than by the degree of lumen stenosis (2, 3), that is, vulnerable carotid plaque can increase the risk of ischemic stroke (4, 5). Atherosclerosis is a chronic inflammatory disease in which immune mechanisms play a pivotal role, and inflammation may promote the occurrence, development, and rupture of plaques (6–8).

It is well known that low-density lipoprotein cholesterol (LDL-C) is associated with cardiovascular disease. However, even though statins can significantly reduce LDL-C levels, which also suppress inflammation (9), cardiovascular risk remains, which may be associated with high levels of inflammation (10, 11). Inflammation is a central driver of atherogenesis and eventual plaque rupture, and it is an important contributor to residual risk. Therefore, based on the high-sensitivity C-reactive protein (hsCRP) level, hsCRP ≥2 mg/L were generally defined as residual inflammatory risk (RIR) (12). It is significantly associated with myocardial infarction, stroke, and all-cause death (13).

To date, the research on RIR mainly focuses on the clinical prognosis of related diseases. Recent studies have reported that persistent high RIR increases the risk of all-cause death and myocardial infarction after percutaneous coronary intervention (PCI) and increases the risk of recurrence in patients with acute ischemic stroke (14, 15). However, there are no reports of high RIR and specific neuroimaging changes. Therefore, this study aimed to investigate the association between high RIR and vulnerable plaques in the carotid artery in patients with acute ischemic stroke.

## Materials and methods

### Study population

Patients with acute ischemic stroke were consecutively enrolled from The First Affiliated Hospital of Anhui University of Science and Technology between January 2021 and July 2022. This study was approved by the ethics committee of The First Affiliated Hospital of Anhui University of Science and Technology, and each patient signed an informed consent form.

The inclusion criteria for this study are as follows: (1) patients had to be aged 18 years or older; (2) have experienced ischemic stroke within the past 7 days; and (3) have undergone completed carotid computed tomography angiography (CTA) scans within 7 days of the index stroke. The exclusion criteria are as follows: (1) had previous carotid endarterectomy or carotid stenting; (2) had active infection; and (3) had malignant tumors or severe heart, liver, or renal failure.

### Baseline data collection

Clinical data including age, sex, height, weight, current smoking and drinking status, medical histories (hypertension, diabetes, atrial fibrillation, coronary heart disease, ischemic stroke, and autoimmune diseases), previous medication (antiplatelet, statins, and anti-inflammatory drug) and National Institutes of Health Stroke Scale (NIHSS) scores were collected from medical records.

### Blood sample collection and measurements

Fasting venous blood was collected the next morning after admission. LDL-C and homocysteine levels were measured using a Siemens ADVIA1800 automatic biochemical analyzer, hsCRP was detected using a Mindray BC7500 automatic blood cell analyzer and leukocyte counts were tested using a Sysmex BC7500 automatic blood routine analyzer.

### Assessment of plaque vulnerability of the carotid artery and the degree of carotid stenosis

Carotid CTA scans were performed with a dual-source 256 spiral CT scanner (Revolution, GE, USA). A non-ionic iodine contrast agent (iodixanol 350–370 mgI/ml) and normal saline were injected through the cubital vein with a total injection volume of 60 ml, and the injection flow rate was 4 ml/s. The monitoring was delayed for 15 s after injection. The scan was started when the contrast medium reached its peak concentration in the target vessel. The scanning parameters were 125 kV tube voltage, 250 mAs tube current, and 512 × 512 matrix. The scan range was from the lower edge of the aortic arch to the cranial roof, and the scan direction was from the foot to the cephalic side. The original data were processed by digital subtraction using CT workstation, and then the carotid arteries were reconstructed, analyzed, and diagnosed by volume reproduction, maximum density projection, multiplane reconstruction, and advanced vascular analysis.

**Figure 1:**
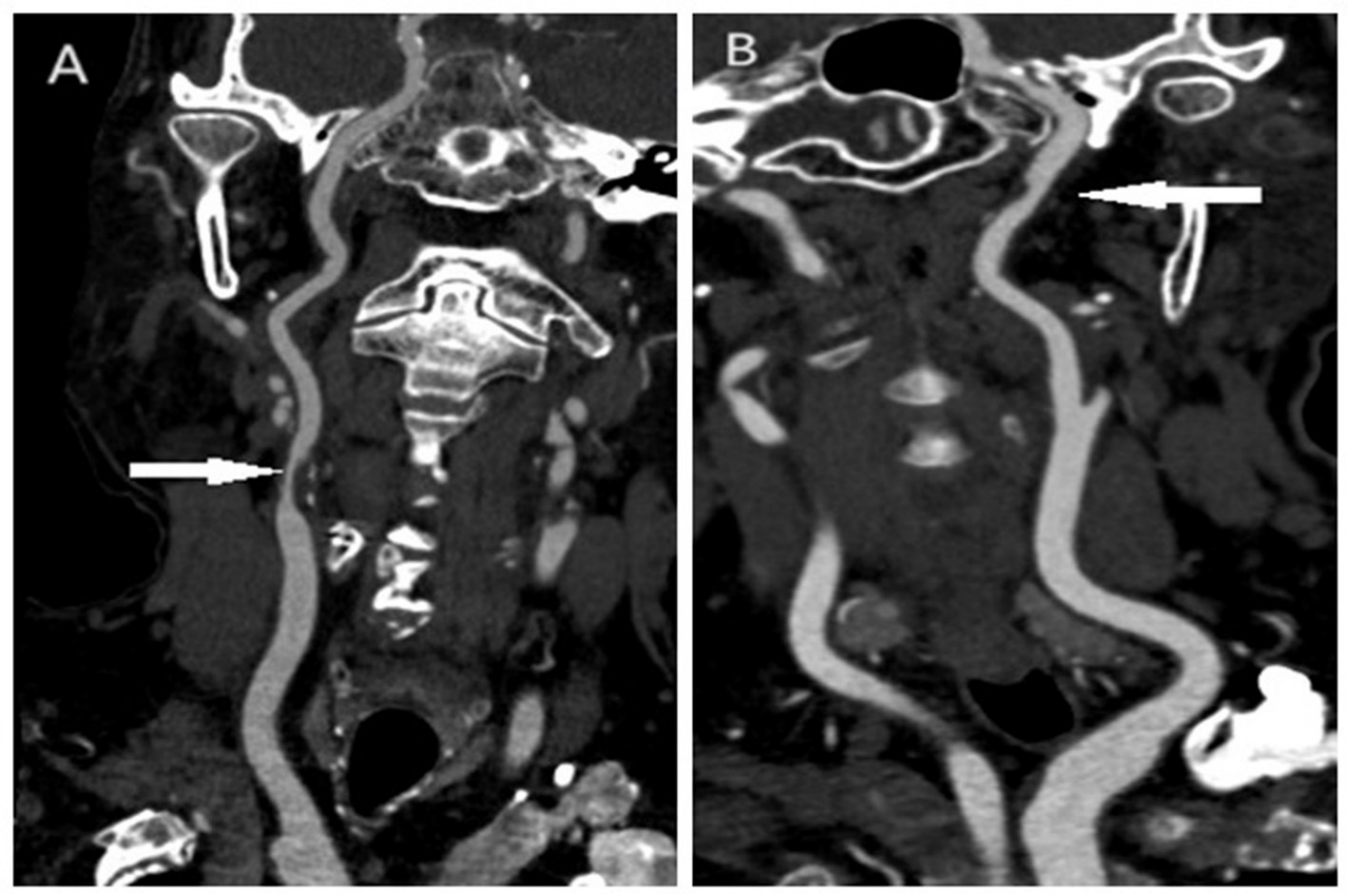
The image of a vulnerable plaque and a non-vulnerable plaque in the carotid artery. **(A)** Vulnerable plaque in the carotid artery; **(B)** non-vulnerable plaque in the carotid artery.

As our previous research showed, vulnerable plaques were determined if low attenuated plaque CT value of < 35 Hounsfield Units (HU) and a remodeling index of >1.1, which indicated a positive remodeling (16). Low attenuated plaque CT value was measured as the minimum CT value of plaque at least three contentiously cross-sectional images at the interest region and averaged; Remodeling index was calculated as the ratio between the outer vessel area (including both plaque and vessel lumen, roughly equals the external elastic membrane area in intravenous ultrasound) at the site of maximal luminal narrowing and the mean of the proximal and distal reference sites. The image of a vulnerable plaque and a non-vulnerable plaque is shown in Figure 1.

According to the degree of carotid artery stenosis, patients were classified into three categories: mild (< 50%), moderate (50%−69%) and severe (≥70%).

### Statistical analysis

SPSS 24.0 (IBM Corp., Armonk, NY, USA) and SAS version 9.4 (SAS Institute Inc., Cary, NC, USA) were used for statistical analysis. The Kolmogorov–Smirnov test was used to analyze whether continuous variables were normally distributed. The data consistent with normal distribution were expressed as mean ± standard deviation, and the comparison between groups was performed using an independent sample *t*-test. The data that were not normally distributed were showed as median and interquartile range, and the contrast between groups was analyzed using the Mann–Whitney *U* test. Categorical variables were expressed as frequency and percentage and the Chi-squared test or Fisher's exact test was used for the comparison between groups.

Generally, an LDL-C level of < 2.6 mmol/L has been recommended as a therapeutic target in high-risk individuals, while a high inflammatory status was defined as hsCRP ≥2 mg/L (17, 18). Therefore, we used an LDL-C level of 2.6 mmol/L and an hsCRP level of 2 mg/L as cut-off values, and the patients were divided into four groups:RIR only (LDL-C < 2.6 mmol/L and hsCRP ≥2 mg/L), residual cholesterol risk (RCR) only (LDL-C ≥2.6 mmol/L and hsCRP < 2 mg/L), both risk or residual cholesterol and inflammatory risk (RCIR) (LDL-C ≥2.6 mmol/L and hsCRP ≥2 mg/L), and neither risk (LDL-C < 2.6 mmol/L and hsCRP < 2 mg/L). The association between residual risk and vulnerable plaques in the carotid artery was assessed using the multiple logistic regression model. Model 1 was adjusted for age and sex. Model 2 was adjusted for factors in model 1 and body mass index, smoking, drinking, medical history of hypertension, diabetes, hyperlipemia, coronary heart disease, atrial fibrillation, ischemic stroke and autoimmune diseases, previous usage of antiplatelet agents, statin and anti-inflammatory drugs, baseline NIHSS scores, and the degree of carotid stenosis. Model 3 was adjusted for factors in model 2 and baseline leukocyte and homocysteine counts. The subgroup analysis was further performed according to the different Trial of ORG 10172 in Acute Stroke Treatment (TOAST) subtypes. The results were expressed as odds ratios (OR) and 95% confidence intervals (CI). A significance level of *P* < 0.05 was considered statistically significant.

## Results

### Baseline characteristics

A total of 756 patients with acute ischemic stroke were enrolled in this study. Among them, 23 (3.0%) patients with previous carotid endarterectomy or carotid stent implantation, 17 (2.2%) patients with active infection, and 42 (5.5%) patients with malignant tumors or severe heart, liver, or kidney failure were excluded. Of the remaining 674 patients, 282 (41.8%) had intracranial arteriosclerotic stenosis, 468 (69.4%) had significant carotid plaque on at least one side and were eventually included in the analysis.

The median age was 64 (55–72) years and 308 (65.8%) of them were male patients. The median levels of LDL-C and hsCRP were 2.53 mmol/L (1.91–3.02 mmol/L) and 1.66 mg/L (1.29–3.14 mg/L). Based on carotid CTA results, vulnerable plaques were present in 157 patients (33.5%).

Table 1 illustrates baseline characteristics with or without vulnerable plaques in the carotid artery. Patients with vulnerable plaques had a higher prevalence of hyperlipidemia (*P* = 0.026), a higher proportion of RIR (*P* = 0.015), a higher ratio of stroke subtypes of large artery atherosclerosis (*P* = 0.012), and high leukocyte counts (*P* < 0.001).

**Table 1:** Baseline characteristics in patients with ischemic stroke.

**Variables**	**Total (*n* = 468)**	**With vulnerable plaque (*n* = 157)**	**Without vulnerable plaque (*n* = 311)**	***P*-value**
Age, years	64.0 (55.0–72.0)	65.0 (56.5–74.0)	64.0 (55.0–72.0)	0.141
Sex, male, *n* (%)	308 (65.8)	110 (70.1)	198 (63.7)	0.168
Body mass index, kg/m^2^	24.0 (22.5–25.6)	24.0 (22.5–25.5)	24.0 (22.5–25.6)	0.555
**Medical history**, ***n*** **(%)**				
Hypertension	283 (60.5)	100 (63.7)	183 (58.8)	0.311
Diabetes	133 (28.4)	44 (28.0)	89 (28.6)	0.893
Hyperlipidemia	43 (9.2)	21 (13.4)	22 (7.1)	0.026
Coronary heart disease	39 (8.3)	14 (8.9)	25 (8.0)	0.745
Atrial fibrillation	43 (9.2)	11 (7.0)	32 (10.3)	0.246
Prior stroke	135 (28.8)	48 (30.6)	87 (28.0)	0.558
Smoking	157 (33.5)	62 (39.5)	95 (30.5)	0.053
Drinking	122 (26.1)	45 (28.7)	77 (24.8)	0.364
Autoimmune diseases	22 (4.7)	9 (5.7)	13 (4.2)	0.454
**Medication history**, ***n*** **(%)**				
Antiplatelet	129 (27.6)	41 (26.1)	88 (28.3)	0.618
Statin	130 (27.8)	43 (27.4)	87 (28.0)	0.894
Anti-inflammatory	19 (4.1)	8 (5.1)	11 (3.5)	0.424
Baseline NIHSS, score	3 (2–4)	4 (2–5)	3 (2–4)	0.112
**The degree of carotid stenosis**, ***n*** **(%)**				
Mild (< 50%)	356 (76.1)	114 (72.6)	242 (77.8)	0.392
Moderate (50%−69%)	80 (17.1)	32 (20.4)	48 (15.4)	
Severe (≥70%)	32 (6.8)	11 (7.0)	21 (6.8)	
**Laboratory data**				
Leukocyte, × 10^9^/L	6.38 (5.20–7.99)	6.94 (5.76–8.71)	6.21 (4.90–7.63)	< 0.001
Homocysteine, μmol/L	11.2 (8.9–14.6)	11.0 (8.9–15.6)	11.2 (9.0–14.4)	0.533
**Groups according to LDL-C and hsCRP levels**, ***n*** **(%)**				
Neither risk	154 (32.9)	37 (23.6)	117 (37.6)	0.015
RIR	134 (28.6)	50 (31.8)	84 (27.0)	
RCR	88 (18.8)	31 (19.7)	57 (18.3)	
RCIR	92 (19.7)	39 (24.8)	53 (17.0)	
**TOAST subtypes**, ***n*** **(%)**				
Large artery atherosclerosis	192 (41.0)	79 (50.3)	113 (36.3)	0.012
Small vessel occlusion	91 (19.4)	28 (17.8)	63 (20.3)	
Others	185 (39.5)	50 (31.8)	135 (43.4)	

NIHSS, National Institutes of Health Stroke Scale; LDL-C, low-density lipoprotein cholesterol; hsCRP, high-sensitivity C-reactive protein; RIR, residual inflammatory risk; RCR, residual cholesterol risk; RCIR, residual cholesterol and inflammatory risk; TOAST, Trial of ORG 10172 in Acute Stroke Treatment.

Table 2 shows baseline characteristics according to LDL-C and hsCRP levels. The proportion of patients with no residual risk, RIR, RCR, and RCIR were 32.9%, 28.6%, 18.8%, and 19.7%, respectively. Hyperlipidemia (*P* = 0.039), baseline leukocyte counts (*P* = 0.010), vulnerable plaques (*P* = 0.015), and stroke subtypes (*P* = 0.003) differed among the four groups.

**Table 2:** Baseline characteristics according to LDL-C and hsCRP levels.

**Variables**	**Neither risk (*n* = 154)**	**RIR (*n* = 134)**	**RCR (*n* = 88)**	**RCIR (*n* = 92)**	***P*-value for trend**
Age, years	62.0 (54.0–70.3)	67.0 (57.0–74.0)	65.5 (55.0–74.0)	65.5 (56.0–71.8)	0.095
Sex, male, *n* (%)	98 (63.6)	89 (66.4)	59 (67.0)	62 (67.4)	0.917
Body mass index, kg/m^2^	24.0 (22.5–25.6)	23.8 (22.4–25.8)	24.6 (22.5–25.5)	23.9 (22.3–25.6)	0.906
**Medical history**, ***n*** **(%)**					
Hypertension	86 (55.8)	83 (61.9)	53 (60.2)	61 (66.3)	0.422
Diabetes	36 (23.4)	40 (29.9)	26 (29.5)	31 (33.7)	0.337
Hyperlipidemia	8 (5.2)	10 (7.5)	13 (14.8)	12 (13.0)	0.039
Coronary heart disease	12 (7.8)	13 (9.7)	8 (9.1)	6 (6.5)	0.838
Atrial fibrillation	11 (7.1)	15 (11.2)	9 (10.2)	8 (8.7)	0.669
Prior stroke	40 (26.0)	48 (35.8)	23 (26.1)	24 (26.1)	0.217
Smoking	47 (30.5)	40 (29.9)	33 (37.5)	37 (40.2)	0.272
Drinking	43 (27.9)	33 (24.6)	23 (26.1)	23 (25.0)	0.925
Autoimmune diseases	8 (5.2)	6 (1.7)	5 (5.7)	3 (3.3)	0.870
**Medication history**, ***n*** **(%)**					
Antiplatelet	40 (26.0)	46 (34.3)	20 (22.7)	23 (25.0)	0.204
Statin	41 (26.6)	47 (35.1)	20 (22.7)	22 (23.9)	0.141
Anti-inflammatory	6 (3.9)	5 (3.7)	5 (5.7)	3 (3.3)	0.852
Baseline NIHSS score	3.5 (2.0–5.0)	3.0 (2.0–4.0)	3.5 (2.0–5.0)	3.0 (2.0–4.0)	0.132
**The degree of carotid stenosis**, ***n*** **(%)**					
Mild (< 50%)	124 (34.8)	101 (28.4)	65 (18.3)	66 (18.5)	0.667
Moderate (50%−69%)	21 (26.3)	24 (30.0)	18 (22.5)	17 (21.3)	
Severe (≥70%)	9 (28.1)	9 (28.1)	5 (15.6)	9 (28.1)	
**Laboratory data**					
Leukocyte, × 10^9^/L	6.30 (5.09–8.04)	6.69 (5.47–7.76)	5.95 (4.77–7.64)	6.78 (5.66–8.99)	0.010
Homocysteine, μmol/L	10.9 (8.9–14.4)	11.3 (9.1–14.8)	12.3 (9.4–16.2)	10.4 (8.5–13.2)	0.089
Vulnerable plaque, *n* (%)	37 (24.0)	50 (37.3)	31 (35.2)	39 (42.4)	0.015
**TOAST subtypes**, ***n*** **(%)**					
Large artery atherosclerosis	47 (30.5)	66 (49.3)	35 (39.8)	44 (47.8)	0.003
Small vessel occlusion	33 (21.4)	18 (13.4)	16 (18.2)	24 (26.1)	
Others	74 (48.1)	50 (37.3)	37 (42.0)	24 (26.1)	

LDL-C, low-density lipoprotein cholesterol; hsCRP, high-sensitivity C-reactive protein; RIR, residual inflammatory risk; RCR, residual cholesterol risk; RCIR, residual cholesterol and inflammatory risk; NIHSS, National Institutes of Health Stroke Scale; TOAST, Trial of ORG 10172 in Acute Stroke Treatment.

### Associations of RCR, RIR, and RCIR with plaque vulnerability

The logistic regression analysis detected that patients with RIR (OR 1.88, 95% CI 1.13–3.13, *P* = 0.015) and RCIR (OR 2.33, 95% CI 1.34–4.05, *P* = 0.003) had a higher risk of vulnerable plaques compared to patients with no residual risk (Table 3). After multivariable adjustment (as in model 3), the association remained (OR 1.98, 95% CI 1.13–3.45, *P* = 0.016; OR 2.14, 95% CI 1.16–3.95, *P* = 0.015; Table 3).

**Table 3:** Logistic regression analysis for exploring the association between RIR, RCR and RCIR with plaque vulnerability.

**Neither risk (*n* = 154)**	**Unadjusted model**		**Model 1**		**Model 2**		**Model 3**	
	**OR (95% CI)**	***P*** **-value**	**OR (95% CI)**	***P*** **-value**	**OR (95% CI)**	***P*** **-value**	**OR (95% CI)**	***P*** **-value**
	Reference		Reference		Reference		Reference	
RIR (*n* = 134)	1.882 (1.131–3.132)	0.015	1.804 (1.080–3.015)	0.024	1.963 (1.144–3.370)	0.014	1.978 (1.134–3.449)	0.016
RCR (*n* = 88)	1.720 (0.970–3.049)	0.064	1.657 (0.931–2.948)	0.086	1.491 (0.812–2.737)	0.198	1.640 (0.876–3.072)	0.122
RCIR (*n* = 92)	2.327 (1.336–4.052)	0.003	2.245 (1.285–3.922)	0.004	2.295 (1.268–4.153)	0.006	2.141 (1.161–3.949)	0.015

Model 1 was adjusted for age and sex; Model 2 was adjusted for factors in model 1 and body mass index, smoking, drinking, medical history of hypertension, diabetes, hyperlipemia, coronary heart disease, atrial fibrillation, ischemic stroke and autoinmmune diseases, previous usage of antiplatelet agent, statin and anti-inflammatory drugs, baseline NIHSS score, and the degree of carotid stenosis; Model 3 was adjusted for factors in model 2 and baseline leukocyte and homocysteine count.

RIR, residual inflammatory risk; RCR, residual cholesterol risk; RCIR, residual cholesterol and inflammatory risk; NIHSS, National Institutes of Health Stroke Scale; OR, odds ratios; CI, confidence interval.

### Associations between RIR and plaque vulnerability based on TOAST classification

**Figure 2:**
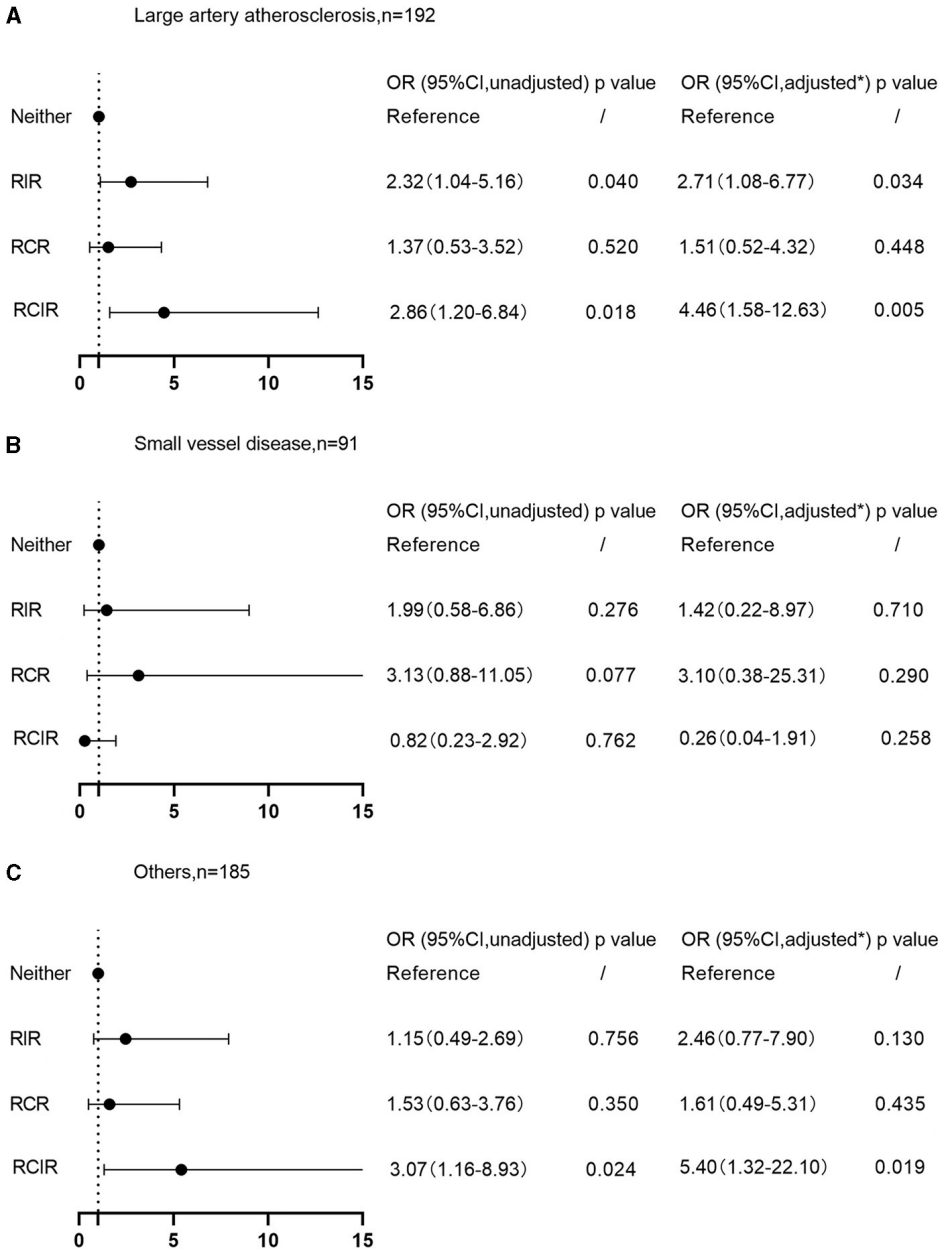
Associations of RIR with plaque vulnerability on TOAST classification. **(A)** Large artery atherosclerosis subtype; **(B)** small vessel occlusion subtype; **(C)** stroke of other subtypes. *Adjusted for age, sex, body mass index, smoking, drinking, medical history of hypertension, diabetes, hyperlipemia, coronary heart disease, atrial fibrillation, ischemic stroke, autoimmune diseases, previous usage of antiplatelet agents, statin and anti-inflammatory drugs, baseline NIHSS scores, the degree of carotid stenosis, and baseline leukocyte and homocysteine counts. RIR, residual inflammatory risk; RCR, residual cholesterol risk; RCIR, residual cholesterol and inflammatory risk; TOAST, Trial of ORG 10172 in Acute Stroke Treatment; NIHSS, National Institutes of Health Stroke Scale; OR, odds ratios; CI, confidence interval.

Further subgroup analysis showed that, after classifying patients according to the TOAST criteria, those with stroke subtypes of large artery atherosclerosis were more likely to develop vulnerable plaques if experiencing RIR (OR 2.32, 95% CI 1.04–5.16 *P* = 0.040; Figure 2A) and RCIR (OR 2.86, 95% CI 1.20–6.84, *P* = 0.018; Figure 2A). After controlling for the potential confounders, results persisted qualitatively similar (OR 2.71, 95% CI 1.08–6.77, *P* = 0.034; OR 4.46, 95% CI 1.58–12.63, *P* = 0.005; Figure 2A). There was no significant interaction between RIR and plaque vulnerability in the patients with small vessel occlusion subtype (OR 1.42, 95% CI 0.22–8.97, *P* = 0.710; Figure 2B) and stroke of other subtypes (OR 2.46, 95% CI 0.77–7.90, *P* = 0.130; Figure 2C).

## Discussion

This study revealed a significant positive association between RIR and vulnerable plaques in the carotid artery in patients with ischemic stroke, especially in the large atherosclerotic subtype, suggesting that these patients may benefit further from initiating anti-inflammatory therapy in addition to lipid-lowering therapy.

Previous research studies have demonstrated that the incidence of RIR varies among different study populations; however, all are so common (19–22). In the large-scale PROVE-IT (Pravastatin or Atorvastatin Evaluation and Infection) study of 3,745 patients with acute coronary syndrome, RIR was present in 29% of those allocated to the atorvastatin 80 mg group (19). Similarly, the IMPROVE-IT (Improved Reduction of Outcomes: Vytorin Efficacy International) trial revealed that, even in patients treated with either simvastatin 40 mg or the combination of simvastatin 40 mg plus ezetimibe 10 mg daily, the proportion of patients with RIR was still as high as 33% (20). In addition, the VIRGO (Variation in Recovery: Role of Gender on Outcomes of Young AMI Patients) registry study of young patients with coronary heart disease has also shown that 60% of patients had elevated hsCRP levels and RIR presented in 16% of the general population (21). The above analysis of individuals with coronary ischemic heart disease revealed that RIR was universal in the real clinical world even when treated with statins. Moreover, in a recent analysis from China's multicenter cohort study of patients with acute ischemic stroke or transient ischemic attack, Li et al. (22) reported that 23.1% of them were afflicted with RIR. However, in the results of our study, the incidence of RIR was 28.6%, which was slightly higher than that reported in Li et al.'s study. This difference may be due to the cutoff point of hsCRP set at 2 mg/L in this study, which is lower than the cutoff value of 3 mg/L in the study of Li et al.

Carotid plaque vulnerability is a suitable indicator to assess the severity of atherosclerosis in the large arteries. The main characteristics of vulnerable plaques include thin fibrous cap, large lipid core, active inflammation, neovascularization, and dilated remodeling. Pathological neovascularization in plaques can promote the development of atherosclerotic lesions and induce intra-plaque bleeding and plaque rupture, which is an important factor causing increased plaque vulnerability (23). RIR may increase the risk of vulnerable plaques in the carotid artery for reasons that are not entirely clear; however, this association may be attributed due to the following reasons: first, hsCRP is one of the most sensitive inflammatory markers, mainly involved in acute non-specific inflammatory responses. Under the stimulation of inflammatory transmitters, it binds to lipoprotein, activates the complement system to produce a large number of terminal complexes, causing vascular endothelial injury, and then exacerbates the inflammatory response in plaques through the release of tissue factors by inflammatory cells to accelerate the formation of intravascular thrombosis (24). Additionally, hsCRP can induce the secretion of matrix metalloproteinases, which increase the fragility of atherosclerotic plaques by accelerating the degradation of endothelial cells (25). Finally, hsCRP can also promote the formation of new blood vessels within the plaque, increasing the risk of bleeding within the plaque and eventually leading to the formation of vulnerable plaques (25). The association between RIR and vulnerable plaques in the carotid artery was only found in patients with large atherosclerosis. This may be because CRP is involved in the formation of atherosclerotic thrombosis which is the pathogenesis of large atherosclerotic cerebral infarction, through a variety of pathways, including activation of the complement system, induction of apoptosis, vascular cell activation, leukocyte recruitment, lipid accumulation, and platelet aggregation (26). Furthermore, CRP also contributes to plaque instability by inducing the expression of metalloproteinases (MMP) 1, 2, and 9 (27, 28). In addition, we also found a significant association between RCIR and carotid plaque vulnerability in the large atherosclerosis subtype, while no such observation was observed with RCR. This further suggests that inflammation plays an important role in promoting the formation of vulnerable plaques in the carotid artery.

There were several limitations in this study. First, this was a single-center study with a relatively small sample size and is not fully representative of the overall population. Second, for hsCRP, we only examined baseline data at admission and did not monitor its dynamic changes as in some other studies (29, 30). Finally, this was a cross-sectional study and, therefore, could not determine cause and effect. However, no cohort studies have been conducted on the association between RIR and vulnerability of plaques. Thus, further prospective studies are needed.

## Conclusion

In patients with acute ischemic stroke, RIR can predict plaque vulnerability in the carotid artery, especially for those with large artery atherosclerosis. Prospective trials should be further explored to investigate inflammation-modulating interventions in these high-risk patients.

## Data availability statement

The raw data supporting the conclusions of this article will be made available by the authors, without undue reservation.

## Ethics statement

The studies involving humans were approved by Ethics Committee of the First Affiliated Hospital of Anhui University of Science and Technology. The studies were conducted in accordance with the local legislation and institutional requirements. The participants provided their written informed consent to participate in this study.

## Author contributions

XG: Writing – original draft, Data curation, Funding acquisition, Investigation, Methodology, Software. CY: Writing – review & editing. ZL: Data curation, Methodology, Writing – original draft. XW: Data curation, Methodology, Writing – original draft. QC: Methodology, Writing – original draft. XC: Methodology, Writing – original draft. JL: Methodology, Writing – original draft.
